# Spontaneous complete resolution of alopecia totalis post SARS-CoV-2 infection

**DOI:** 10.1016/j.jdcr.2022.07.027

**Published:** 2022-08-03

**Authors:** Georgio Chidiac, Remie Chrabieh, Micheline Maamari, Jinane El Khoury, Nakhle Ayoub

**Affiliations:** a Faculty of Medicine and Medical Sciences, Holy Spirit University of Kaslik (USEK), Jounieh, Lebanon; b Department of Dermatology, University Hospital Center-Notre Dame des Secours, Byblos, Lebanon; c Department of Dermatology, Lebanese American University Medical Center-Rizk Hospital, Beirut, Lebanon; d Department of Dermatology, Sacré Cœur Hospital, Baabda, Lebanon; e Department of Dermatology, Rose Marie and Gilbert Chaghoury School of Medicine, Lebanese American University, Beirut, Lebanon; f Department of Dermatology, French Hospital of the Levant, Beirut, Lebanon

**Keywords:** alopecia areata, alopecia totalis, COVID-19, JAK inhibitor, SARS-CoV-2, tofacitinib, AA, Alopecia areata, AT, Alopecia totalis, JAK, Janus kinase

## Introduction

COVID-19, linked to the infection with SARS-CoV-2, has been associated with a plethora of cutaneous manifestations with an estimated prevalence ranging from 0.2% to 20% according to various studies.^1^ Review articles and case series have documented several types of skin eruptions including erythematous macules and papules, urticarial, papulosquamous, varicelliform, morbilliform, purpuric, livedo reticularis–like, retiform purpura, petechial, and pernio-like acro-ischemic lesions.^1^ Hair and nail involvement has additionally been reported such as telogen effluvium and leukonychia. Furthermore, the course of many immune-mediated diseases, including dermatological conditions such as alopecia areata (AA), might be affected by COVID-19.^2^ We herein report an unusual case of alopecia totalis (AT) recovery occurring after a SARS-CoV-2 infection.

## Case report

**Fig 1:**
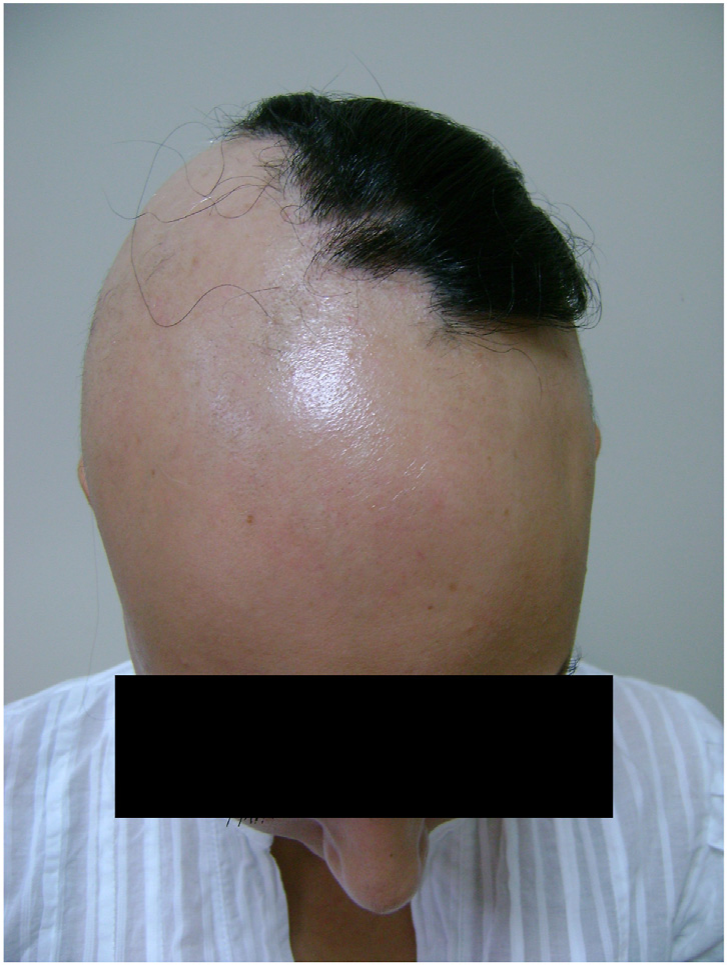
Patient with partial hair regrowth while on methotrexate.

**Fig 2:**
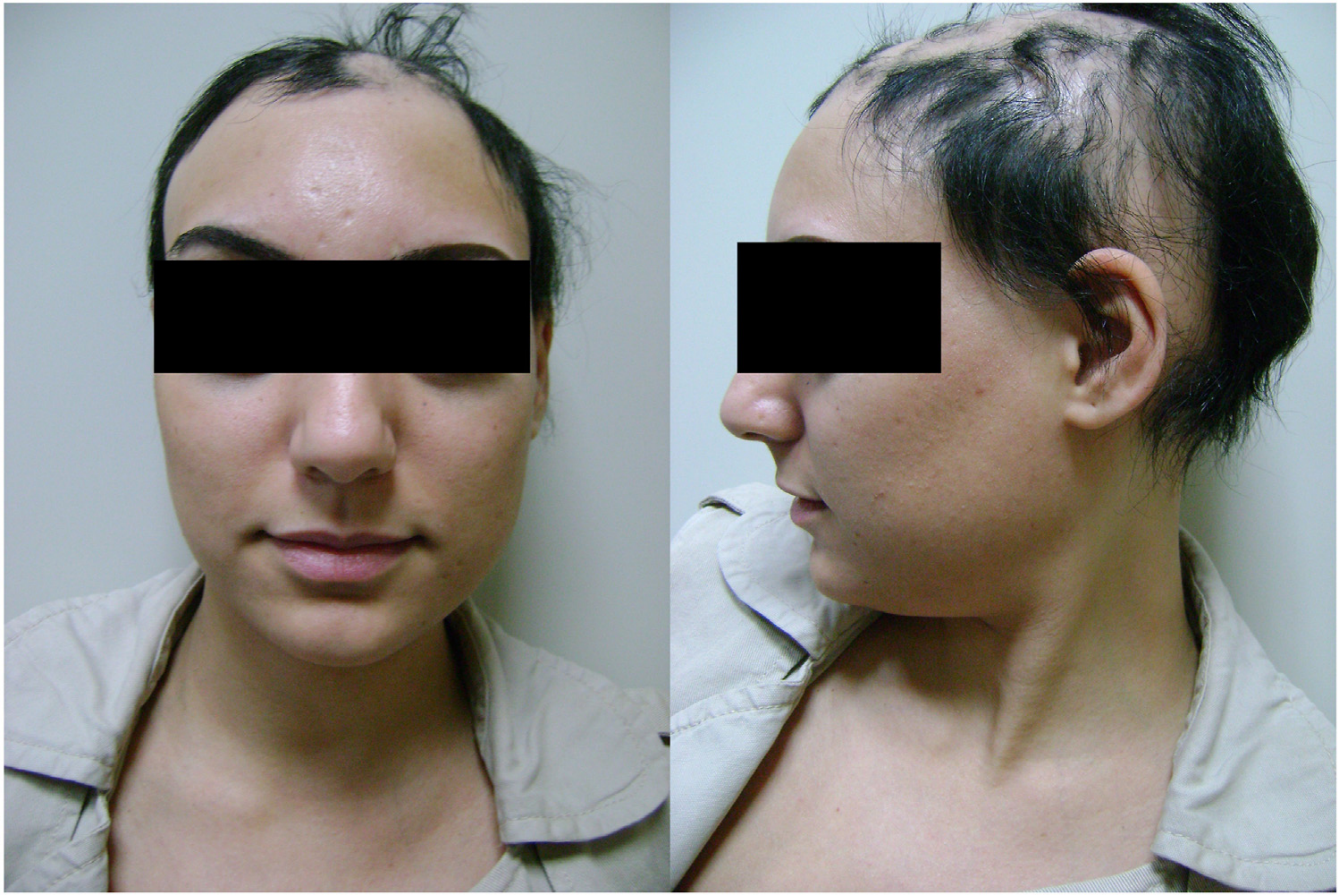
Patient with patchy hair regrowth while using tofacitinib 15 mg daily.

**Fig 3:**
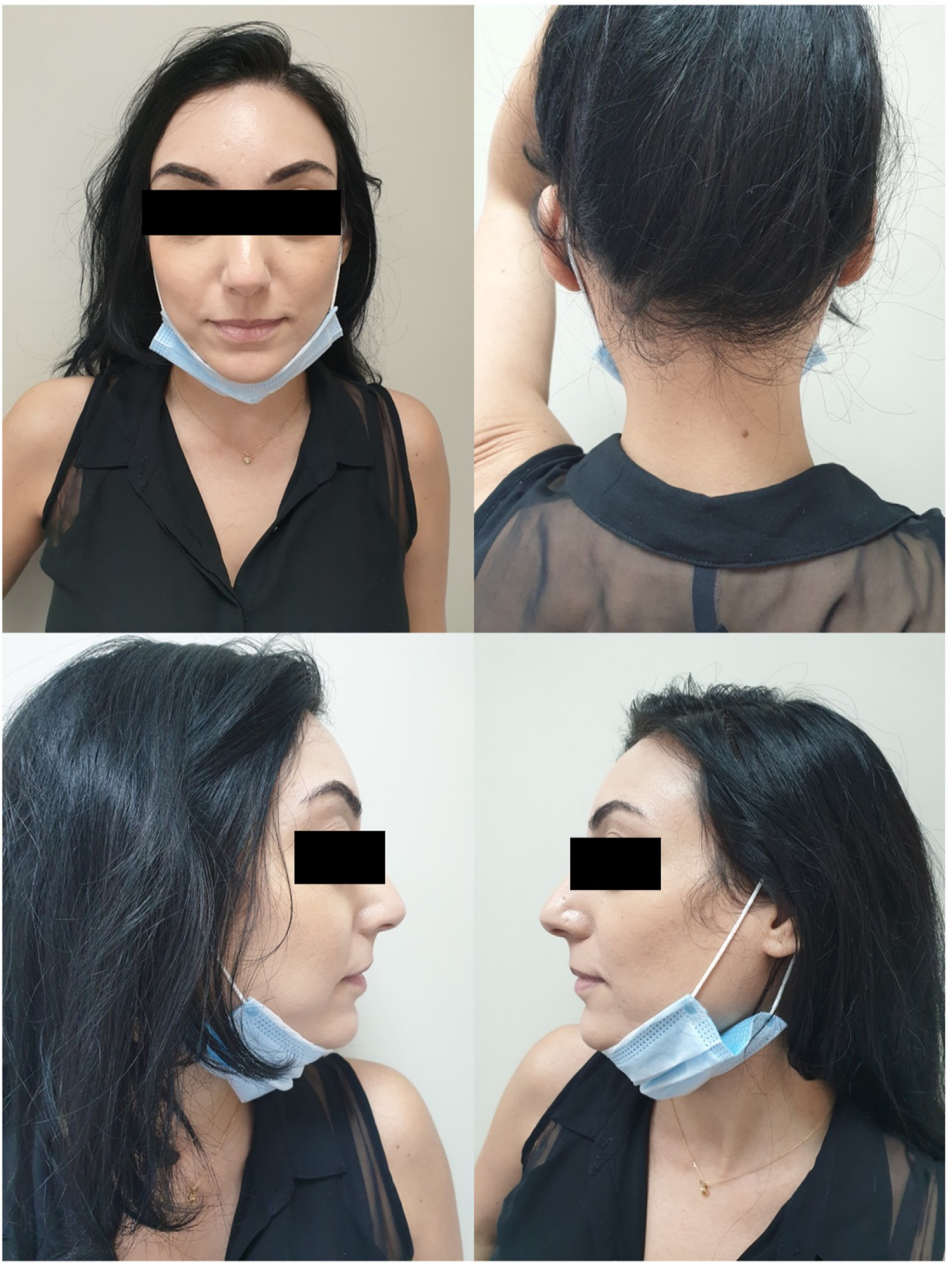
Full hair regrowth after recovering from COVID-19.

A 32-year-old female patient had had AT for 13 years. She had received during the course of her illness several therapeutic agents including topical, oral, intravenous, and intralesional corticosteroids; topical minoxidil 5% solution; and oral isoprinosine, all of which resulted in poor response. Partial hair regrowth was observed with hydroxychloroquine and methotrexate (Fig 1). She was started on tofacitinib alone with a 15-mg daily dose in 2017 and reported additional improvement but never achieved full hair regrowth (Fig 2). However, in the early days of the COVID-19 pandemic, the patient discontinued her medication due to fear of the immunomodulating effects of tofacitinib. Shortly after stopping her medication, she started experiencing hair shedding. In October 2020, the patient tested positive for SARS-CoV-2. The diagnosis was made by real-time reverse transcriptase polymerase chain reaction test of nasopharyngeal swabs. She was unvaccinated against COVID-19 and experienced during her illness mild symptoms of fever, body ache, and cough. She had no shortness of breath and did not require hospital admission. Interestingly, 3 months after recovering from COVID-19 and while she had been off tofacitinib for 10 months, a spontaneous total hair regrowth started to be observed, and satisfying results were obtained approximately a year later (Fig 3). However, she regressed shortly afterward with a recurrence of her AT and lost all her scalp hair over a 3-month period. In the meanwhile, the patient did not get reinfected with COVID-19, nor did she get vaccinated because of vaccine skepticism and despite our advice.

## Discussion

AA is a complex autoimmune disorder that attacks the hair follicle epithelium causing nonscarring hair loss. The exact etiology remains elusive, but numerous factors have been suggested as possible triggers in genetically predisposed individuals, such as stress, viruses, hormonal alterations, and vaccination.^3^

Several cases of new-onset AA developing after COVID-19 have been reported, but it is not clear whether theses cases were a consequence of the psychological distress related to the infection, the inflammatory state associated with COVID-19, or a possible effect of the virus on the hair follicle immune privilege.^4^ The infection, however, could play a role in the worsening of a pre-existing AA, occurring more likely in patients who discontinue their treatment.^5^ Alternatively, mild-to-moderate COVID-19 does not seem to affect the course of the disease in patients who remain on active AA treatment.^6^ Moreover, COVID-19 vaccines may play a role in the activation of different immunological events leading to an aberrant autoimmune response that could trigger AA in susceptible individuals.^7^

At the beginning of the pandemic, and due to the unknown course of the disease, patients were initially advised to withhold immunosuppressive medications, including Janus kinase (JAK) inhibitors, during active SARS-CoV-2 infection. Thereafter, multiple cases of patients with AA who were kept on tofacitinib treatment during their active COVID-19 infection were reported not to have a worse outcome while on JAK inhibitors.^5^^,^^8^ Moreover, JAK inhibitors are currently being investigated as potential therapeutic alternatives for severe COVID-19 infection. The continuous use of JAK inhibitors in AA patients who develop COVID-19 infection is still controversial. However, it has been suggested that patients should be counseled about the relative reported safety of continuing JAK inhibitors during COVID-19 to limit AA progression.^9^

There is no report of full hair regrowth after COVID-19 in patients with pre-existing AA. However, one case report describes some improvement after a SARS-CoV-2 infection in a 27-year-old female known to have alopecia universalis successfully treated with tofacitinib and who maintained her treatment during her disease.^8^ One possible mechanism to explain the full hair regrowth seen in our patient might be that SARS-CoV-2 inhibits the autoimmune reaction against hair follicles by antigenic competition, a phenomenon in which the immune response to one antigen is suppressed by the response to another unrelated antigen.^10^ In our case, we speculate that COVID-19 may have induced hair regrowth by playing the role of the second antigen that prevented the immune system from attacking hair follicles. However, we cannot rule out the possibility that the 2 events were coincidental since AA patients may experience spontaneous remission during the course of their disease, albeit much less in the case of AT.

In conclusion, we present a case of complete hair regrowth post COVID-19 in a patient known to have AT. Our unique observation in the setting of a large-scale worldwide pandemic may help raise awareness concerning a possible shift in immunity induced by COVID-19 in specific individuals. This report provides, in our opinion, a contribution to the current knowledge about the presumptive association between SARS-CoV-2 infection and transient AA recovery.

## Conflicts of interest

None disclosed.
